# Latent classes and predictors of aggression trajectories in Korean adolescents: Implications for targeted prevention

**DOI:** 10.1371/journal.pone.0354136

**Published:** 2026-07-16

**Authors:** Eunha Jeong

**Affiliations:** School Health Teacher, School Health Clinic, Doonchon Middle School, Seoul, Republic of Korea; Jawaharlal Institute of Postgraduate Medical Education and Research, INDIA

## Abstract

School violence and juvenile crime are emerging social problems in Korea. Adolescent aggression, an important mediator linking risk factors in the developmental environment to more serious deviant behavior, exerts long-term cumulative effects into adulthood. Thus, a longitudinal examination of aggression among adolescents is crucial. This study identified differential longitudinal trajectories of aggression in Korean adolescents and investigated the predictors distinguishing these latent classes. Data were used from 2,016 adolescents from Waves 1–5 (2018–2022) of the Korean Children and Youth Panel Survey. The overall trajectory of adolescent aggression was explored using latent growth curve modeling, and latent class growth modeling was conducted to identify possible heterogeneity in aggression trajectories. Multinomial logistic regression was used to analyze the predictors of each latent class. Latent growth curve modeling revealed an overall decline in aggression across adolescents. Latent class growth modeling identified three latent classes—moderate-decreasing, low-increasing, and high-decreasing—indicating heterogeneous developmental patterns of adolescent aggression. Key predictors of latent class included gender, perceived economic status, impulsivity, depression, smartphone dependency, negative parenting attitude, and negative peer relationships. Adolescent aggression follows heterogeneous developmental pathways shaped by individual, family, and school factors. These findings highlight the importance of identifying distinct aggression trajectory groups and their key predictors and demonstrate the value of a trajectory-based approach to guide targeted prevention strategies.

## Introduction

In 2023, the school violence perpetration rate in Korea was 1.9%, and the victimization rate was 1.0%, reflecting a slight increase compared to 2022 (0.2% and 0.4%, respectively) [1]. The proportion of juvenile offenders involved in heinous crimes, such as murder and rape, among all arrested criminals in Korea also increased from 7.9% in 2015 to 9.6% in 2021 [2]. Moreover, as the scope of youth crime has expanded from the physical to the digital world, the number of adolescent cybercrime suspects has surged from 8,642 in 2018–12,165 in 2020 [3]. School violence and juvenile crimes are not unique to Korea. In the US, bullying is a prominent concern [4]: Although rates of traditional offline bullying have stabilized, cyberbullying is rising dramatically [5]. Juvenile crime is also an ongoing national problem [6]. As school violence and youth crime become pressing social problems, researchers are increasingly focusing on the development of youth aggression, which is considered a crucial mediating factor linking risk factors in the developmental environment to subsequent, more serious deviant behavior [7,8].

Aggression is a broad concept that can be classified into various types depending on its form of expression, such as overt or direct aggression, when it is expressed verbally and physically, and relational aggression, which is expressed indirectly through exclusion or damage to peer relationships; or by function including proactive or instrumental aggression, which involves attacking others to satisfy one’s needs, and reactive aggression, which occurs as a defensive response to perceived aggression [7,8]. Aggression is inherent and can be expressed in different ways, with major change patterns and degrees occurring in adolescence [9]. As an important predictor of more serious deviant or antisocial behavior in adulthood [10], adolescent aggression warrants attention.

Adolescent aggression has long-term cumulative effects on psychosocial functioning and can influence both potential criminal behavior and success in adulthood [11]. From a developmental perspective, aggression is not a static trait but evolves over time through interactions between individual characteristics and environmental influences, reflecting key principles of developmental psychopathology [7–9]. To better understand this, this study conducted a longitudinal examination of aggression in Korean adolescents. Previous studies investigating changes in aggression in Korean adolescents have certain limitations, such as relying on cross-sectional comparisons of specific time periods, using panel data [12], or exploring changes based on a single developmental trajectory applied to all individuals [13–15]. These studies have drawn inconsistent conclusions, with some finding that aggression increases over time [13,15] and others reporting a decrease [12,14]. Such variability likely results from treating the entire adolescent population as a homogeneous group. Recent trajectory-based approaches have highlighted that adolescent aggression does not follow a single developmental pattern but instead consists of heterogeneous pathways that differ in onset, stability, and change over time [13–15]. Thus, this study employed latent class growth analysis to distinguish between different developmental trajectories of adolescent aggression within this population.

Factors explaining individual differences in aggression range from genes to socialization [8,16]. Since Bronfenbrenner introduced the ecological systems theory in 1977 [17], scholars have emphasized that adolescents’ aggression should be interpreted within the broader system that affects its development [18]. In other words, effective interventions to prevent the development of aggression should consider not only the microsystem that has direct contact with adolescents but also the mesosystem (the relationships between components of the microsystem), exosystem (external influences that affect development), and macrosystem (cultural and societal values) [8,18]. Ecological systems theory provides a comprehensive framework for understanding how such multilevel influences contribute to heterogeneous aggression trajectories across adolescence [8,17,18]. Factors known to affect youth aggression include gender [12,19], household economic status [20], self-esteem [15], impulsivity [21], depression [21], smartphone dependency [22], parenting attitudes [14,15], peer relationships [23,24], and relationships with teachers [24]. However, most studies have focused either on the relationship between aggression and a limited number of predictors or on comparisons among variable-centered individuals. In particular, there is a lack of research that simultaneously examines heterogeneous aggression trajectories and their multilevel predictors within a unified analytical framework, highlighting a critical gap in the literature. Therefore, this study aimed to (1) identify the longitudinal trajectories of adolescent aggression, (2) examine whether heterogeneous latent classes of aggression exist, and (3) determine which individual and contextual factors predict membership in these trajectories.

## Materials and methods

### Study design

This study conducted a secondary data analysis using longitudinal data from the Korean Children and Youth Panel Survey (KCYPS) 2018 provided by the National Youth Policy Institute Youth and Children Data Archive (https://www.nypi.re.kr/archive/eps). It aimed to classify latent classes based on trajectories of adolescent aggression and identify the predictive factors of these classes.

### Participants

This study used data from Waves 1–5 (2018–2022) of the KCYPS 2018, a longitudinal study conducted with cohorts extracted using multistage stratified cluster sampling, with first-year middle-school students nationwide as the target population. Data were collected using tablet PC-assisted individual face-to-face interviews, with adolescent participants and their parents interviewed separately in accordance with the standardized KCYPS protocol. At baseline (Wave 1, 2018), the mean age of participants was 12.99 years (SD = 0.13), corresponding to the first year of middle school in Korea. At Wave 5 (2022), the mean age was 16.99 years (SD = 0.13), corresponding to the second year of high school. A dataset was constructed by integrating annual data from 2018 to 2022 using household and personal IDs. Of the original 2,590 panel members, 2,016 participated in all five waves and had complete data across the study variables. These were the participants included in the final analyses. The National Youth Policy Institute applies standardized panel management procedures, including annual follow-up tracking and non-response-adjusted sampling weights, to maintain the representativeness of the KCYPS cohort. These weighting procedures were designed to minimize the potential bias arising from differential dropout across waves. This study was approved by the Public Institutional Review Board of the Ministry of Health and Welfare (IRB No. P01-202406-01-015). The requirement for informed consent was waived because this study used secondary data. The data were accessed for research purposes between June 16 and 30, 2024.

### Measurements

All variables were measured using validated scales included in the KCYPS 2018. For each construct, detailed information on the scale source, number of items, scoring procedure, and internal consistency (Cronbach’s α) is provided as follows.

### Dependent variable

Six items related to aggression were taken from the Emotional and Behavioral Problem Scale [25] included in the KCYPS 2018. The selected items assess adolescents’ aggressive tendencies, expressed through anger, confrontational responses, and conflict-related behaviors. Each item was measured on a 4-point Likert scale ranging from “not at all” (1 point) to “very much” (4 points), with a higher score indicating a higher level of aggression. Cronbach’s α was .83, .85, .85, .85, and .83 in 2018, 2019, 2020, 2021, and 2022, respectively. Internal consistency was stable across all five waves, indicating acceptable reliability of the aggression measure at each assessment.

### Independent variables

Sociodemographic factors included gender (boy/girl), average monthly household income, perceived economic status, and parental education. Average monthly household income was rated on a 12-point scale ranging from no income (1 point) to over 10 million Korean won (KRW) (12 points). Perceived economic status was rated on a 5-point scale from “lowest” (1 point) to “highest” (5 points), with a higher score indicating higher perceived economic status. Parental education level ranged from “uneducated” (1 point) to “master’s or doctoral degree” (7 points) and was calculated as the average of both parents’ educational levels. If data were available for only one parent, that parent’s education level was used.

Individual factors included self-esteem, impulsivity, depression, and smartphone dependency. For self-esteem, 10 items from the Self-Esteem Scale [26] were used. Each item was rated on a 4-point Likert scale ranging from “not at all” (1 point) to “very much” (4 points). Five negatively worded items were reverse-scored, with a higher total score indicating higher self-esteem. Cronbach’s α was .87 in this study. Impulsivity was investigated using seven items related to impulsivity from the Emotional and Behavioral Problem Scale [25]. Each item was rated on a 4-point Likert scale ranging from “not at all” (1 point) to “very much” (4 points), with a higher total score indicating higher impulsivity. Cronbach’s α was .82 in this study. For depression, 10 items from the Symptom Checklist-90-Revised were used [27]. Each item was measured on a 4-point Likert scale ranging from “not at all” (1 point) to “very much” (4 points), with a higher score indicating a higher level of depression. Cronbach’s α was .92 in this study. Smartphone dependency was analyzed using adolescents’ responses to the Smartphone Addiction Proneness Scale [28]. The scale comprised 15 items, and each item was rated on a 4-point Likert scale ranging from “not at all” (1 point) to “very much” (4 points). Among the 15 items, three—“Smartphone use does not interfere with the work (study) I am doing now,” “I do not feel anxious even without a smartphone,” and “I do not spend much time using a smartphone”—were reverse-coded, meaning that a higher score indicates higher smartphone dependency. Cronbach’s α was .88 in this study. The present study used the smartphone dependency measure as per the standardized KCYPS 2018 protocol for adolescent participants, following the original survey design.

Family factors included parenting attitudes. This was assessed using 24 items from the Korean version of the Parents as Social Context Questionnaire for Adolescents [29]. This scale comprises three subfactors of positive parenting attitudes (autonomy support, warmth, and structure provision) and three subfactors of negative parenting attitudes (rejection, coercion, and inconsistency). Each item was rated on a 4-point Likert scale, with responses ranging from “not at all” (1 point) to “very much” (4 points); a higher total score on the 12 positive or 12 negative items indicated stronger positive or negative parenting attitudes, respectively. In this study, Cronbach’s α coefficients for positive and negative parenting attitudes were .92 and .87, respectively.

School factors included peer and student–teacher relationships. Peer relationships were measured using the Peer Relationship Quality Scale for Adolescents [30]. This scale comprises 13 items: eight items for positive peer relationships and five for negative peer relationships. Each item was rated on a 4-point Likert scale ranging from “not at all” (1 point) to “very much” (4 points), with a higher total score indicating a stronger perception of positive or negative peer relationships. In this study, Cronbach’s α coefficients were .89 and .73 for positive and negative peer relationships, respectively. The student–teacher relationship was assessed using 14 items from the Student–Teacher Attachment Relationship Scale [31]. Each item was rated on a 4-point Likert scale ranging from “not at all” (1 point) to “very much” (4 points), with a higher total score indicating a better relationship with the teacher. Cronbach’s α was .91 in this study.

### Data analysis

An unconditional latent growth curve model was incorporated to investigate the overall trajectory of adolescent aggression between 2018 and 2022. Model fit was verified using the complementary fit index, Tucker–Lewis index, and root mean square error of approximation (RMSEA). The best model fit criteria were 0.95 or more for the complementary fit and Tucker–Lewis Index, and 0.06 or lower for the RMSEA [32]. As the analytic dataset contained no missing values across study variables, no additional imputation procedures were required.

Latent class growth modeling, which produces clearly distinguishable classes, was conducted to model the possible heterogeneity in aggression trajectories. Latent Class Growth Analysis (LCGA) was selected because the primary objective of this study was to identify distinct developmental trajectory groups with relatively homogeneous within-class patterns and to examine their associated predictors. Analyses were conducted using robust standard errors, which helped reduce potential bias related to the clustered sampling design. The optimal number of latent classes was determined according to the Akaike information criterion, Bayesian information criterion, adjusted Bayesian information criterion, Lo–Mendell–Rubin adjusted likelihood ratio test, bootstrapped likelihood ratio test, entropy, and the minimum class size (at least 5% of participants in each class). For the information-based goodness-of-fit indices (Akaike information criterion, Bayesian information criterion, and adjusted Bayesian information criterion), smaller values indicate better model fit [33]. Significant *p*-values in the Lo–Mendell–Rubin adjusted likelihood ratio test and bootstrapped likelihood ratio test suggest that the model with *k* classes is more applicable than the *k*-1 class model [34]. Entropy values closer to 1 indicate better classification accuracy, with 0.6 or higher recommended as the minimum acceptable value [33]. The final number of latent classes was selected based on a comprehensive evaluation of these criteria, including model fit indices, likelihood ratio tests, entropy, and theoretical interpretability.

Finally, multinomial logistic regression was conducted to identify predictive factors for the latent classes. Descriptive statistics, independent sample *t*-tests, Pearson’s correlation analysis, and multinomial logistic regression analysis were performed using SPSS/WIN 29.0 (IBM Institute, NY, USA). Multicollinearity diagnostics indicated no evidence of problematic multicollinearity among the predictors (VIFs = 1.01–2.16; tolerance values = 0.46–0.99). Latent growth curve modeling and latent class growth modeling were conducted using Mplus 8.11 (Muthen & Muthen, CA, USA).

## Results

### Participant characteristics and changes in aggression

The descriptive statistics and correlation coefficients of the main variables are presented in Table 1. Among all participants, 53.1% (*n* = 1,071) were boys and 46.9% (*n* = 945) were girls. Significant gender differences were observed in the mean aggression scores in 2018. Girls reported slightly higher aggression scores than boys (Student’s t = 3.26, p = .001). Levene’s test supported the assumption of homogeneity of variance (F = 3.37, p = .066), and the effect size was very small (Cohen’s d = 0.15), indicating limited practical significance. All sociodemographic-, individual-, family-, and school-related factors were correlated with aggression in 2018. Mean aggression scores were 11.48 ± 3.51 in 2018, 11.26 ± 3.53 in 2019, 11.11 ± 3.55 in 2020, 11.03 ± 3.38 in 2021, and 11.27 ± 3.34 in 2022.

**Table 1:** Characteristics of the sample (*N* = 2,016).

Variables	Categories	*n* (%) or M ± SD	Observed range	Aggression (2018)	
M ± SD	*t* or *r* (*p*)
Sociodemographic factors					
Gender	Boy	1,071 (53.1)		11.24 ± 3.41	3.26 (.001)
	Girl	945 (46.9)		11.75 ± 3.61	
Average monthly household income		6.62 ± 2.27	1–12		−0.11 (< .001)
Perceived economic status		2.95 ± 0.53	1–5		−0.09 (< .001)
Parental education level		5.07 ± 0.96	1–7		−0.11 (< .001)
Individual factors					
Self-esteem		29.97 ± 5.08	11–40		−0.46 (< .001)
Impulsivity		15.15 ± 3.96	7–28		0.60 (< .001)
Depression		18.00 ± 6.41	10–40		0.58 (< .001)
Smartphone dependency		30.55 ± 7.34	15–58		0.42 (< .001)
Family factors					
Positive parenting attitudes		39.22 ± 5.77	14–48		−0.35 (< .001)
Negative parenting attitudes		23.82 ± 6.34	12–48		0.45 (< .001)
School factors					
Positive peer relationships		24.98 ± 4.22	8–32		−0.21 (< .001)
Negative peer relationships		9.25 ± 2.60	5–20		0.45 (< .001)
Student–teacher relationship		39.11 ± 7.00	14–56		−0.31 (< .001)
Dependent variable					
Aggression	2018	11.48 ± 3.51	6–24		
	2019	11.26 ± 3.53	6–24		
	2020	11.11 ± 3.55	6–24		
	2021	11.03 ± 3.38	6–24		
	2022	11.28 ± 3.34	6–24		

M, mean; SD, standard deviation

### Trajectories of adolescent aggression

Longitudinal trajectory analysis of participants’ aggression from 2018 to 2022 revealed an initial mean intercept of 11.36 (*p* < .001) and a mean slope of −0.06 (*p* = .007); thus, both were statistically significant. Both the complementary fit and Tucker–Lewis indices were 0.95 or higher, and the RMSEA was 0.06 or lower, indicating that the model fit the data well (Table 2).

**Table 2:** Trajectory of aggression among adolescents (*N* = 2,016).

Latent variables	Mean	SE	*p*	χ^2^ (*p*)	CFI	TLI	RMSEA
Initial intercept	11.36	0.07	< .001	66.62 (< .001)	0.969	0.969	0.05
Slope	−0.06	0.02	.007				

SE, standard error; CFI, comparative fit index; TLI, Tucker–Lewis index; RMSEA, root mean error of approximation.

### Latent classes based on adolescent aggression trajectories

Table 3 presents the results of the latent class analyses for models with two to five latent classes. Overall, the fit indices supported a three-class model, as indicated by lower values for the Akaike information criterion, Bayesian information criterion, and adjusted Bayesian information criterion when comparing the two- and three-class models. Additionally, the Lo–Mendell–Rubin adjusted likelihood ratio test and bootstrapped likelihood ratio test results were significant, and the higher entropy value indicated greater classification accuracy for the three- versus the four-class model. The three-class model was ultimately selected based on fit, interpretability, and parsimony.

**Table 3:** Goodness-of-fit statistics for the latent class models by number of classes (*N* = 2,016).

Model	Model fit index						*n* (%) according to latent class				
AIC	BIC	Adjusted BIC	LMR-LRT *p*-value	BLRT *p*-value	Entropy	Class 1	Class 2	Class 3	Class 4	Class 5
2-class	51,858.37	51,931.28	51,889.98	.005	< .001	0.425	902 (44.8)	1,113 (55.2)			
**3-class**	**51,834.25**	**51,923.99**	**51,873.16**	**.003**	**< .001**	**0.622**	**1,004 (49.8)**	**689 (34.2)**	**323 (16.0)**		
4-class	51,820.72	51,927.29	51,866.92	.037	< .001	0.527	466 (23.1)	603 (29.9)	729 (36.2)	218 (10.8)	
5-class	51,831.79	51,955.19	51,885.29	.998	.999	0.632	134 (6.7)	749 (37.1)	44 (2.2)	632 (31.8)	458 (22.7)

AIC, Akaike information criterion; BIC, Bayesian information criterion; LMR-LRT, Lo–Mendell–Rubin adjusted likelihood ratio test; BLRT, bootstrapped likelihood ratio test. Bolded model is the selected one.

**Fig 1:**
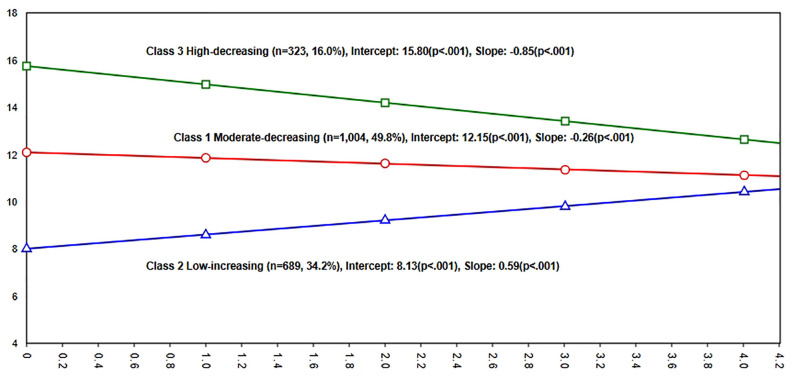
Latent classes of aggression trajectories.

Fig 1 illustrates the three distinct classes identified according to adolescents’ aggression trajectories. Class 1 was named a “moderate-decreasing” class because aggression began with a moderate initial value (intercept: 12.15, *p* < .001), and the slope decreased slightly (slope: −0.26, *p* < .001). Class 1 accounted for 49.8% (*n* = 1,004) of the participants. Class 2, a “low-increasing” class, began with a low initial value of aggression (intercept: 8.13, *p* < .001) and the slope increased slightly (slope: 0.59, *p* < .001). Class 2 accounted for 34.2% (*n* = 689) of the participants. Class 3, a “high-decreasing” class, began with a high initial value of aggression (intercept: 15.80, *p* < .001), and the slope decreased (slope: −0.85, *p* < .001). Class 3 accounted for 16.0% (*n* = 323) of the participants.

### Predictors of each latent class

Table 4 presents the results of the multinomial logistic regression analysis using Class 2 (low-increasing) as the reference group. The multinomial logistic regression model was statistically significant (χ² = 1040, df = 26, p < .001). The Cox and Snell R² and Nagelkerke R² values were 0.159 and 0.331, respectively, indicating a moderate level of explanatory power. The model deviance (−2 Log Likelihood) was 2893. First, the higher the impulsivity (odds ratio [OR] = 1.204, 95% confidence interval [CI] = 1.160–1.249), the higher the depression (OR = 1.115, 95% CI = 1.085–1.146), smartphone dependency (OR = 1.028, 95% CI = 1.009–1.048), negative parenting attitude (OR = 1.030, 95% CI = 1.005–1.056), negative peer relationships (OR = 1.143, 95% CI = 1.082–1.207), and likelihood of being classified into the moderate-decreasing class compared to the reference group. Second, the more girls than boys (OR = 1.727, 95% CI = 1.187–2.512), the lower the perceived economic status (OR = 0.665, 95% CI = 0.453–0.976) and the higher the impulsivity (OR = 1.444, 95% CI = 1.361–1.532), depression (OR = 1.196, 95% CI = 1.150–1.244), smartphone dependency (OR = 1.048, 95% CI = 1.019–1.077), negative parenting attitude (OR = 1.071, 95% CI = 1.034–1.110), negative peer relationships (OR = 1.335, 95% CI = 1.0233–1.446), and likelihood of being classified into the high-decreasing class in comparison with the reference group.

**Table 4:** Multinomial logistic regression.

Variables	Class 1 (Moderate decreasing)		Class 3 (High decreasing)	
	OR	(95% CI)	OR	(95% CI)
Gender (ref. boy)	1.071	(0.834–1.376)	**1.727**	**(1.187–2.512)**
Average monthly household income	1.001	(0.939–1.068)	0.990	(0.898–1.092)
Perceived economic status	0.826	(0.632–1.081)	**0.665**	**(0.453–0.976)**
Parental education level	1.037	(0.905–1.189)	1.008	(0.822–1.236)
Self-esteem	1.003	(0.969–1.038)	0.994	(0.945–1.045)
Impulsivity	**1.204**	**(1.160–1.249)**	**1.444**	(1.361–1.532)
Depression	**1.115**	**(1.085–1.146)**	**1.196**	(1.150–1.244)
Smartphone dependency	**1.028**	**(1.009–1.048)**	**1.048**	(1.019–1.077)
Positive parenting attitude	0.984	(0.957–1.011)	0.982	(0.944–1.021)
Negative parenting attitude	**1.030**	**(1.005–1.056)**	**1.071**	(1.034–1.110)
Positive peer relationships	1.003	(0.971–1.036)	1.031	(0.982–1.082)
Negative peer relationships	**1.143**	**(1.082–1.207)**	**1.335**	(1.233–1.446)
Student–teacher relationship	0.992	(0.972–1.013)	0.988	(0.959–1.018)

Reference = Class 2 (low increasing); ref., reference; OR, odds ratio; CI, confidence interval; bold text indicates significance.

## Discussion

The data used in this study were well-suited for the linear model, as indicated by the latent growth curve modeling, which showed a steady decrease in aggression among Korean adolescents over the five-year period from the first year of middle school to the second year of high school (intercept 11.36; slope −0.06). Although the mean aggression score showed a slight increase from 2021 to 2022, the overall five-year pattern remained downward. One possible explanation is that this modest rebound may reflect contextual changes during the later phase of the COVID-19 pandemic, including the gradual resumption of in-person school activities and peer interactions. However, because only one post-2022 observation was available, this interpretation remains speculative and should be examined in future longitudinal studies. These results are consistent with those of prior studies on the developmental trajectory of aggression among Korean adolescents. Lee et al. [35], who examined students from the second year of middle school to the first year of high school, and Han and Kim [14], who examined students from the sixth year of elementary school to the first year of high school, showed that aggression significantly decreased over time. One reason could be that as the age of secondary school students increases, their physical, cognitive, and emotional maturity increases, and they can better control their aggression [12]. Additionally, as adolescents spend more time at school than at home, parental attachment decreases, they receive less supervision [13], and increasingly focus on peer relationships [35].

By contrast, aggression has been reported to increase in early adolescence, particularly in Grades 4–6 of Korean elementary school [13,15]. These results can be interpreted as an inflection point that turns from increasing aggression to a decreasing trend at the time of the transition from early to middle adolescence. This study used five years of data from a first-year middle-school cohort because the KCYPS 2018 provides data only up to 2022. Future research should aim to create an aggression development model using a wider range of long-term data from elementary to middle school to develop aggression mitigation programs tailored to different stages of adolescence in Korea.

Second, the latent class growth modeling analysis revealed that the trajectory of aggression was divided into three latent classes, despite the decrease in aggression among all Korean adolescents. The three latent classes were classified as the moderate-decreasing group (49.8%), in which moderate aggression gradually decreased over time; the low-increasing group (34.2%), which initially started with low aggression and increased over time; and the high-decreasing group (16.0%), in which the initial high aggression decreased over time. In comparison, Seo and Kim [19] classified changes in aggression into three types—intermediate-level stable (71.2%), increased (24.0%), and decreased (4.8%)—for early adolescents from the fourth grade of elementary school to the first grade of middle school. Similarly, Kim [36] divided the developmental trajectory of aggression from late childhood to late adolescence into three subgroups: moderate maintenance (83.3%), decreased (9.9%), and increased (6.8%). Despite the difficulty of direct comparisons owing to participants’ different age groups, this study showed a decrease in the proportion of the moderate-decreasing group but an increase in that of the high-decreasing and low-increasing groups compared to prior studies. Differences in the identified trajectory patterns may also reflect methodological differences across studies, including differences in the trajectory modeling approach. This finding suggests the importance of screening and early intervention for high-risk groups for aggression. In addition to primary prevention-oriented emotional and behavioral intervention programs for all adolescents, intervention strategies for high-risk groups need to be developed.

However, Ha’s [37] study from the fourth grade of elementary school to the first grade of high school classified the latent classes of aggression into three types: medium-to-small decrease group (52.8%), low-decrease group (33.7%), and high-increase group (13.5%). Although these aforementioned studies all focused on Korean adolescents, the inconsistent results may be due to different sample sizes, study scales, and timing of cohort formation. Therefore, this study’s use of the most recent KCYPS 2018 panel data to examine five-year changes in youth aggression provides added value. This is because longitudinal study results based on empirical data, such as developmental trajectories and the number and characteristics of latent classes, can shift depending on the sample size or cohort formation timing.

Third, the multinomial logistic regression analysis identified impulsivity, depression, smartphone dependency, negative parenting attitudes, and negative peer relationships as major predictors of latent classes of adolescent aggression. Compared to the reference group with the lowest aggression, adolescents with higher impulsivity showed elevated depression and greater smartphone dependency, experienced more negative parenting attitudes and negative peer relationships, and were more likely to be included in the moderate- and high-decreasing aggression groups. This study contributes to the literature by verifying the predictive factors affecting aggression trajectories in various domains—individuals, families, and schools—among Korean adolescents.

Impulsivity was the most significant predictive factor for categorizing the latent classes in this study. This finding is supported by existing studies that have verified the association between impulsivity and aggression in adolescents [21,38]. Impulsivity is a personality trait characterized by complex interactions between genetic and environmental factors. A close relationship exists between the increased possibility of impulsivity in adolescent behavior and neurodevelopmental processes during adolescence [21]. To explain the causal mechanisms of impulsivity and aggression, Mobini et al. [39] suggested that a lack of response inhibition, which causes quick and unprepared responses, eventually results in cognitive distortion, leading to a different interpretation of a given situation (e.g., personalization, negative bias, overgeneralization, and blame). Impulsivity has been shown to be associated with mental disorders, such as antisocial personality disorder and substance abuse, as well as domestic violence and violations of traffic laws [40]. Impulsivity and its adverse consequences are major public health concerns. Early intervention is crucial, particularly considering the longitudinal and bidirectional relationship between impulsivity and aggression [38].

The individual factors of depression and smartphone dependency were also significant predictors of differences between latent classes. Depression, an internalizing problem, and aggression, an externalizing problem, occur simultaneously in adolescence [41]. Studies have found that adolescent depression and aggression have a mutual causal relationship and are highly likely to cause negative synergy [42,43]. Similar to the adjustment erosion hypothesis, adolescent emotional behavior problems may adversely affect other developmental consequences [44]. Specifically, ample evidence shows that the reciprocal relationship between aggression and depression is linked to poorer adjustment among adolescents in multiple domains, such as academic failure, lack of social skills, and substance use [42].

Similar to the results of this study, Lee [22] reported that adolescents with higher smartphone dependency showed higher initial levels of aggression, while aggression decreased over time. This pattern can be understood from a developmental perspective, which suggests that externalizing behaviors such as aggression tend to decline across adolescence [9]. In this context, smartphone dependency may be associated with underlying emotional vulnerability, particularly depressive symptoms, which contribute to higher initial levels of aggression. Supporting this interpretation, previous research has shown that smartphone addiction is dynamically related to aggression, with state-level smartphone addiction predicting subsequent aggression and depression partially mediating this relationship [45]. Taken together, these findings suggest that smartphone dependency may function not as a stable causal factor but as an indicator of short-term emotional dysregulation that elevates initial aggression but does not necessarily sustain aggressive behavior over time.

Another factor distinguishing the low-increasing latent class from the other classes was negative parenting attitude. This finding is consistent with previous studies showing that higher levels of parental abuse are associated with higher initial levels of adolescent aggression and that changes in parental abuse are positively related to changes in aggression over time [14,19]. From a social learning perspective, repeated exposure to coercive, rejecting, or inconsistent parenting may reinforce aggressive behavioral patterns by modeling maladaptive responses to conflict and emotion regulation [46]. Moreover, within an ecological framework, negative parenting attitudes can be understood as proximal processes in the family microsystem that continuously shape adolescents’ emotional and behavioral regulation [18]. Such environments may increase emotional instability and vulnerability, thereby contributing to higher-risk aggression trajectories. Taken together, these findings suggest that negative parenting attitudes are more critical than positive parenting in shaping aggression trajectories, highlighting the importance of reducing coercive parenting practices and strengthening adaptive parenting strategies to prevent the development of aggressive behavior.

Compared with the aggression reference group (low-increasing class), negative peer relationships, a school factor, significantly increased the likelihood of belonging to the moderate- and high-decreasing classes. Kim and Nho [23] reported a longitudinal reciprocal relationship between adolescent peer relationship difficulties and aggressive behavior, emphasizing that the influence of peer relationship difficulties on aggressive behavior is much greater than the effect of aggressive behavior on peer relationship difficulties. During adolescence, peer relationships have an important influence on adaptation, and adolescents regard friends as a crucial source of social relationships [47]. Positive socialization can strengthen bonds with society, lowering the probability of engaging in antisocial behaviors, such as delinquency [48]. Additionally, social information processing bias has been suggested to mediate the relationship between antecedents such as peer rejection and subsequent aggressive development [16]. Therefore, an intervention strategy to improve the quality of peer relationships is recommended to reduce adolescent aggression.

Among the sociodemographic factors, gender and perceived economic status were the main factors that increased the likelihood of belonging to the high-decreasing class compared to the low-increasing class, which was the reference group. Girls were more likely to belong to the group with higher initial aggression levels. This finding should be interpreted in light of the characteristics of the study population and the specific aggression construct assessed. In Sung and Kim’s [49] study comparing gender differences in aggression over three years, from the second year of middle school to the first year of high school, girls had a higher level of aggression than that boys. It is widely known that gender differences are influenced by the type of aggression, with overt aggression being more common in boys and relational aggression in girls. However, research findings vary depending on the definition of aggression, participants, and measurement tools [50]. Therefore, the observed gender difference should be interpreted within the context of the specific aggression construct assessed in this study. Empirical research has demonstrated that girls who show direct aggression have a greater frequency and intensity of aggression and are more likely to develop psychosocial problems than boys [50]. Furthermore, this study showed that lower economic status was associated with a higher likelihood of belonging to the high-decreasing class than to the reference group (low-increasing class). This finding is supported by Shameem and Hamid [20], who reported a negative association between socioeconomic status and aggression, and Kim [36], who reported differences in the latent classes of developmental trajectories of aggression between poverty and non-poverty groups.

Taken together, these findings can be interpreted within Bronfenbrenner’s ecological systems framework [17], highlighting that adolescent aggression develops through the interaction of multiple levels of influence [18]. Rather than reflecting isolated effects, individual vulnerabilities such as impulsivity, depression, and smartphone dependency operate in conjunction with contextual factors, including negative parenting practices and peer relationship difficulties. These results underscore the importance of considering how proximal environments shape behavioral development through ongoing interactions across ecological systems. Accordingly, interventions should adopt a multilevel approach that simultaneously addresses individual regulation, family dynamics, and peer contexts to prevent and reduce adolescent aggression more effectively.

### Limitations and future research directions

This study has several limitations that should be considered. First, although longitudinal data were used, the observed associations should not be interpreted as strictly causal relationships. Future research should further examine the mechanisms underlying these associations using designs better suited to causal inference. Second, this study relied on self-report measures, which may be subject to reporting bias or social desirability effects. Future studies should incorporate multi-informant or objective measures to enhance the validity of the findings. Third, more diverse variables were not included in predicting the risk factors for each latent class of aggression using specific panel data. In subsequent studies, contextual factors, such as community characteristics or culture, that correspond to the exo- or macro-systems of ecological system theory should be included [8,18]. Fourth, the use of complete-case analysis may have introduced potential attrition or selection bias, as only participants with complete data across all waves were included. Future research should consider analytical approaches that address missing data and potential bias. In addition, predictor analyses were conducted using the most likely latent class membership derived from the LCGA. Although this conventional approach has been widely used in previous trajectory studies, more advanced three-step procedures explicitly account for classification uncertainty and may provide less biased estimates of the associations between predictors and latent class membership. Future research may benefit from applying these methods to further evaluate the robustness and generalizability of the present findings. Finally, this study only used data on Korean youth, which hinders the generalizability of the findings to other countries and age groups. Korean youth are located within a unique Korean context comprising an extremely competitive educational environment and collectivist culture, which affects other forms of aggression, delinquency, and bullying [1,4]. Therefore, future studies should focus on other cultures and countries, and comparative studies of other countries, including Korea, should be conducted.

### Implications

First, the study’s methodology expands the existing longitudinal research approaches by moving beyond traditional analyses of average trajectories and variance between individuals. Using the latest large-scale longitudinal data at the national level, this study identifies distinct developmental trajectories of aggression. Second, this study provides useful information for guiding interventions to prevent aggression and facilitate positive changes. Aggression prevention programs may benefit from adopting multilateral interventions not only at the individual level but also at the environmental level, focusing on complex factors such as impulsivity, depression, smartphone dependency, parenting attitudes, and friendships. The identified latent trajectory groups may also assist in the early identification of adolescents at elevated risk for persistent aggression, enabling schools and community mental health services to provide timely and targeted interventions. Since aggression was associated with multiple individual, family, and school factors, prevention strategies may be strengthened by integrating emotional regulation support, parent-focused education, and peer relationship enhancement rather than relying on single-component interventions.

## Conclusions

This study demonstrated that adolescent aggression follows heterogeneous developmental trajectories rather than a single uniform pattern, identifying three distinct groups: moderate-decreasing, low-increasing, and high-decreasing. Individual factors (impulsivity, depression, and smartphone dependency) and contextual factors (negative parenting attitudes and peer relationship difficulties) were important predictors distinguishing these trajectories. These findings highlight the importance of early identification of adolescents at elevated risk for persistent aggression and provide evidence supporting multilevel intervention strategies that address individual, family, and peer contexts.
